# Adrenergic β_2_-Receptors Mediates Visceral Hypersensitivity Induced by Heterotypic Intermittent Stress in Rats

**DOI:** 10.1371/journal.pone.0094726

**Published:** 2014-04-14

**Authors:** Chunhua Zhang, Yun-Yun Rui, Yuan-Yuan Zhou, Zhong Ju, Hong-Hong Zhang, Chuang-Ying Hu, Ying Xiao, Guang-Yin Xu

**Affiliations:** 1 Department of Gastroenterology, the Second Affiliated Hospital, Soochow University, Suzhou, P. R. China; 2 Jiangsu Key Laboratory of Translational Research and Therapy for Neuro-Psycho-Diseases, Institute of Neuroscience, Department of Neurobiology, Soochow University, Suzhou, P. R. China; Zhejiang University School of Medicine, China

## Abstract

Chronic visceral pain in patients with irritable bowel syndrome (IBS) has been difficult to treat effectively partially because its pathophysiology is not fully understood. Recent studies show that norepinephrine (NE) plays an important role in the development of visceral hypersensitivity. In this study, we designed to investigate the role of adrenergic signaling in visceral hypersensitivity induced by heterotypical intermittent stress (HIS). Abdominal withdrawal reflex scores (AWRs) used as visceral sensitivity were determined by measuring the visceromoter responses to colorectal distension. Colon-specific dorsal root ganglia neurons (DRGs) were labeled by injection of DiI into the colon wall and were acutely dissociated for whole-cell patch-clamp recordings. Blood plasma level of NE was measured using radioimmunoassay kits. The expression of β_2_-adrenoceptors was measured by western blotting. We showed that HIS-induced visceral hypersensitivity was attenuated by systemic administration of a β-adrenoceptor antagonist propranolol, in a dose-dependent manner, but not by a α-adrenoceptor antagonist phentolamine. Using specific β–adrenoceptor antagonists, HIS-induced visceral hypersensitivity was alleviated by β_2_ adrenoceptor antagonist but not by β_1_- or β_3_-adrenoceptor antagonist. Administration of a selective β_2_-adrenoceptor antagonist also normalized hyperexcitability of colon-innervating DRG neurons of HIS rats. Furthermore, administration of β-adrenoceptor antagonist suppressed sustained potassium current density (*I*_K_) without any alteration of fast-inactivating potassium current density (*I*_A_). Conversely, administration of NE enhanced the neuronal excitability and produced visceral hypersensitivity in healthy control rats, and blocked by β_2_-adrenoceptor antagonists. In addition, HIS significantly enhanced the NE concentration in the blood plasma but did not change the expression of β_2_-adrenoceptor in DRGs and the muscularis externa of the colon. The present study might provide a potential molecular target for therapy of visceral hypersensitivity in patents with IBS.

## Introduction

Irritable bowel syndrome (IBS), a common functional gastrointestinal disorder, is defined by recurrent symptoms of abdominal pain or discomfort associated with alterations in bowel habits [1]. Up to date, its cardinal feature, chronic visceral pain, has been difficult to treat effectively. Much progress has been made during last several decades in understanding the development of visceral hypersensitivity, but its pathophysiology is not fully understood. Clinical studies demonstrate that chronic stress plays an important role in the pathophysiology of IBS [2]–[4]. Alterations in adrenergic signaling have been implicated in the development of visceral hypersensitivity [5]–[7]. It is reported that chronic stress may induce abnormal expressions of brain G proteins, colonic alpha (2A)-adrenoceptors, and norepinephrine (NE) reuptake transporter (NET), which may be responsible for the abnormalities of abdominal sensation in IBS [8]. Heterotypic chronic stress can increase sympathetic nervous system activity and induces the release of NE [5]. Once released, NE binds to its receptors. The receptors for NE are a class of G protein-coupled receptors, including α and β subtypes. α receptors have α_1_ and α_2_ subtypes while β receptors have β_1_, β_2_ and β_3_ subtypes. β_1_ and β_2_ adrenergic receptors involve in the adrenergic activation of electrogenic K^+^ secretion in guinea pig distal colonic epithelium [9], which may play a role in colonic transit. β_2_ adrenergic receptors located on primary afferent nociceptors are reported to produce a hyperalgesic state in rats [10]–[12]. β_3_ adrenergic receptors, mainly expressed in brown and white adipose tissue, can regulate energy metabolism and thermogenesis [13]. Previous study showed that the blockade of α_1_/α_2_- and β_1_/β_2_-adernoreceptors before the daily application of chronic stress prevented the induction of visceral hypersensitivity in male Wistar rats [6]. However, which subtype of the adrenergic receptors involves in the induction of visceral hypersensitivity following chronic stress remains unclear.

Visceral hypersensitivity has merged as a key hypothesis or biological hallmark in explaining the painful symptoms in IBS [14]–[16]. Both the central sensitization and peripheral sensitization have been implicated in the development of visceral hypersensitivity [17], [18]. The peripheral sensitization involves an enhanced excitability of primary afferent nociceptors, which are the information superhighway from the gut to the spinal cord and convey peripheral stimuli into action potentials that propagate to the central nervous system [17]. Sensitization of primary sensory afferents is maintained by a number of membrane receptors and ion channels. Voltage-gated potassium (Kv) channels play a fundamental role in controlling neuronal excitability. Suppression of K currents contributes to peripheral sensitization [19]. The transient A-type current (*I*_A_) and the sustained delayed rectifier current (*I*_K_) are two primary types of Kv currents that are important in modulating spike frequency and activation thresholds. The decrease in *I*_A_ currents took part in enhanced excitability of pancreatic DRG neurons observed in chronic pancreatitis [20]. The decrease in *I*_K_ took part in colonic DRGs of neonatal maternal separation rats [21] and contributed to hyperexcitability of colonic DRG neurons in mouse. Taken together, the changes in Kv channel function in DRG neurons are disease/organ specific. However, the role of Kv channels in HIS-induced visceral hypersensitivity remains unknown.

In this study, we investigated the contribution of different subtypes of adrenergic receptors to visceral hypersensitivity and to changes in the membrane properties and excitability of colon innervating DRG neurons in a rat model of visceral hypersensitivity induced by heterotypical intermittent stress (HIS). Furthermore, we specifically examined changes in Kv channel conductance in these cells. We tested the hypothesis that enhanced release of NE in response to HIS acts through activation of β_2_-adrenoreceptor to suppress *I*_K_ and sensitize the colon-specific DRG neurons, thus induces visceral hypersensitivity to CRD. The present study suggests that adrenergic receptors are potential treatment targets for visceral pain in patents with IBS.

## Materials and Methods

### Animals and heterotypic intermittent stress protocol

Adult male Sprague-Dawley (SD) rats, weighing 250±20 g, were obtained from the Animal Research Center of Soochow University. Rats were housed under controlled conditions (07:00∼19:00 lighting, temperature: 24±2°C) with free access to a standard laboratory diet and fresh water. Care and handling of these rats were approved by the Institutional Animal Care and Use Committee of the Soochow University and were in accordance with the guidelines of the International Association for the Study of Pain (IASP). Visceral hypersensitivity was induced by heterotypic intermittent stress (HIS), as described previously [6], [22]. In brief, rats were subjected to an HIS protocol composed of 3 randomly arranged stressors: 45 minutes of cold restraint stress at 4°C, 60 minutes of water avoidance stress, or 20 minutes of forced swimming stress for 9 consecutive days.

### Measurement of visceromoter response to graded colorectal distention

Visceral hypersensitivity was measured by grading the response of rats to colorectal distention (CRD) as described previously [23]–[25]. In brief, rats were lightly sedated with diethyl ether while a flexible balloon (6 cm) made of a surgical glove finger attached to a tygon tubing was inserted 8 cm into the descending colon and rectum via the anus and held in place by taping the tubing to the tail. SD rats were placed in small lucite cubicles and allowed to adapt for 30 mins. CRD was performed by rapidly inflating the balloon to a constant pressure measured using a sphygmomanometer connected to a pressure transducer. The balloon was inflated to various pressures: 20, 40, 60 and 80 mmHg, for a 20 sec stimulation period followed by a 2 mins rest. Behavioral responses to CRD were measured by visual observation of the abdominal withdrawal reflex (AWR) by a blinded observer and the assignments of an AWR score were as follows [23]–[25]: 0 =  Normal behavior without response; 1 =  Brief head movement at the onset of the stimulus followed by immobility; 2 =  Contraction of abdominal muscles; 3 =  Lifting of the abdomen off the platform; 4 =  Body arching and lifting of pelvic structures. In addition, colonic distension threshold, the minimal pressure to induce abdominal muscle contraction, was used to measure the time course of HIS and drug effects.

### Cell labeling

Colon-innervating DRG neurons were labeled by injection of 1, 1′-dioleyl-3, 3, 3′, 3-tetramethylindocarbocyanine methanesulfonate (DiI, Invitrogen) into the colon wall as described previously [25]. Briefly, 6-week-old rats were anaesthetized with ketamine (80 mg/kg, i. p.) plus xylazine (5∼10 mg/kg, i. p.). The abdomen was opened by midline laparotomy and the colon was exposed. DiI was injected in ∼1 µl volume (25 mg in 0.5 ml methanol) at 10 sites on the exposed colon extending from the level of the bladder to ∼6 cm in an oral direction. To prevent possible contamination of adjacent organs with the dye, the needle was left in place for ∼1 min and the injection site was washed with normal saline (NS) after each injection. The colon was gently swabbed prior to closing of the abdomen. Rats were returned to their housing and given free access to drinking water and standard food pellets. Ten days later, these rats were sacrificed for whole-cell patch clamp recordings.

### Whole-cell patch clamp recordings

As described previously [25], DRGs (T_13_-L_2_) were dissected out and incubated in dissecting solution with enzymes (collagenase D, 1.5∼1.8 mg/ml, Roche; trypsin, 1.2 mg/ml, Sigma) for 1.5 hours at 34.5°C. DRGs were then taken from the enzyme solution, washed, and transferred to 2 ml of dissecting solution containing DNase (0.5 mg/ml, Sigma). Single cell suspension was obtained by repeat trituration through flame-polished glass pipettes. Coverslips containing adherent DRG cells were put in a small recording chamber (∼1 ml volume) and attached to the stage of an inverted microscope (Olympus IX71, Japan) fitted for both fluorescence and bright-field microscopy. DiI-labeled neurons were identified by their fluorescence under the fluorescent microscope. Neuronal activities were sampled at 10 KHz and filtered at 2 or 5 KHz. Action potential (AP) and voltage-gated potassium currents were recorded with whole-cell patch clamp techniques by a patch-clamp amplifier (HEKA Elektronik, Lambrecht, GER). Data were stored for offline analysis. For patch-clamp recording experiments, normal external solution contained: 130 mM NaCl, 5 mM KCl, 2 mM KH_2_PO4, 2.5 mM CaCl_2_, 1 mM MgCl_2_, 10 mM HEPES, 10 mM glucose (pH = 7.2–7.3 adjusted with NaOH; osmolarity = 295–300 mOsm). Recording pipettes were pulled from borosilicate glass tubing using a horizontal puller (P-97, Sutter Instruments) and had a resistance of 3∼5 MΩ when filled with normal pipette solution containing (in mM): 140 KCl, 10 NaCl, 5 EGTA, 1 CaCl_2_, 10 HEPES, 10 glucose (pH = 7.25 adjusted with KOH; osmolarity = 306 mOsm). For recording potassium currents, pipette solution contained (in mM): 140 KCl, 1 CaCl_2_, 2 MgCl_2_, 9 EGTA, 0.3 GTP, 10 HEPES, (pH = 7.3 adjusted with KOH; osmolarity = 285–295 mOsm), while bath solution contained (in mM): 150 choline chloride, 5 KOH, 0.03 CaCl_2_, 10 HEPES, 3 Mg(OH)_2_, 10 glucose (pH = 7.4 adjusted with HCl; osmolarity  = 310 mOsm).

### Drug application

For behavioral, whole-cell patch clamp and protein experiments, phentolamine (Phen), propranolol (Prop), butoxamine (Buto), atenolol (Aten), SR-59230A (SR) or normal saline (NS) was intraperitoneally (i.p.) administrated 30 minutes before each stressor starting from the 1st day to the 9th day during the HIS protocol. Thus, antagonists or NS were injected for 9 consecutive days. AWR scores were tested 6 hours after termination of the last stressor. Animals were euthanized 6 hours after the last stressor and the colon innervating DRG neurons were harvested for whole-cell patch clamping. In some experiments, butoxamin was intraperitoneally (i.p.) administrated 30 min before NE injection intrathecally. For *in vitro* patch clamp recording study, NE at the concentration of 10 µM was used to incubate the acutely isolated DRG neurons for 3 minutes.

### Western blotting

DRGs (T_13_-L_2_) and the muscularis externa of the distal colon from HIS-treated rats or age-mateched controls were dissected out and lyzed in radioimmunoprecipitation assay buffer containing 1% NP-40, 0.5%Na deoxycholate, 0.1% SDS, PMSF (10 µl/ml), and aprotinin (30 µl/ml; Sigma). The lysates were then microfuged at 15,000 rpm for 30 minutes at 4°C. The concentration of protein in homogenate was determined using a BCA reagent (Beyotime, CHN). Twenty micrograms (20 µg) of proteins for β_2_-adernoceptor studies were loaded onto a 10% Tris-HCl SDS-PAGE gel (Bio-Rad, Hercules, CA). After electrophoresis, the proteins were electrotransferred onto polyvinyldifluoride membranes (Millipore) at 200 mA for 2 hours at 4°C. The membranes were incubated in 25 ml of blocking buffer (1XTBS with 5% w/v fat-free dry milk) for 2 hours at room temperature. The membranes were then incubated with the primary antibodies for 2 hours at room temperature. Primary antibodies used were mouse anti-β_2_-adrenoreceptor (1∶100; abcam, USA) and mouse anti-actin (1∶1000; Chemicon, Temecula, CA) or anti-GAPDH (1∶1000; Goodhere, China). After incubation, the membranes were washed with TBST (1XTBS and 5% Tween 20) three times for 15 minutes each and incubated with anti-mouse HRP-conjugated secondary antibody (1∶4000; Chemicon) for 2 hours at room temperature. The membrane was washed with TBST three times for 15 minutes each. The immunoreactive proteins were determined by enhanced chemiluminescence (ECL kit; Amersham Biosciences, Arlington Heights, IL). Bands were visualized by exposure of membranes onto an x-ray film. For quantification of β_2_-adernoceptor protein levels, photographs were digitalized and analyzed using a scanner (Bio-Rad imaging system Bio-Rad GelDoc XRS+). β-actin or GAPDH was used as an internal control. All samples were normalized to β-actin or GAPDH.

### Measurement of norepinephrine (NE) in blood plasma

Blood samples were collected from the trunk in tubes containing 2.5% sodium citrate and 0.45% citric acid at the time of animal euthanasia by decapitation. Samples from HIS and age-matched control rats were spun in a refrigerated centrifuge; plasma was quickly aliquoted and stored at −80°C for assays. Plasma level of NE was measured using the radioimmunoassay kits from Abnova.

### Data analysis

All data obtained are expressed as mean±SEM in the present study. Statistical analysis were performed using commercial software OriginPro 8 (OriginLab, US). Normality was checked before analyses. Significance was determined using paired sample t-test, paired sample sign test, Mann-Whitney test, Tukey post hoc test following Kruskal-Wallis ANOVA or one-way ANOVA, Dunn's post hoc test following Friedman ANOVA, two sample t-test, as appropriate. The level of significance was set at *p*<0.05.

## Results

### HIS-induced visceral hypersensitivity was attenuated by β-adrenoceptor antagonist propranolol

**Figure 1:**
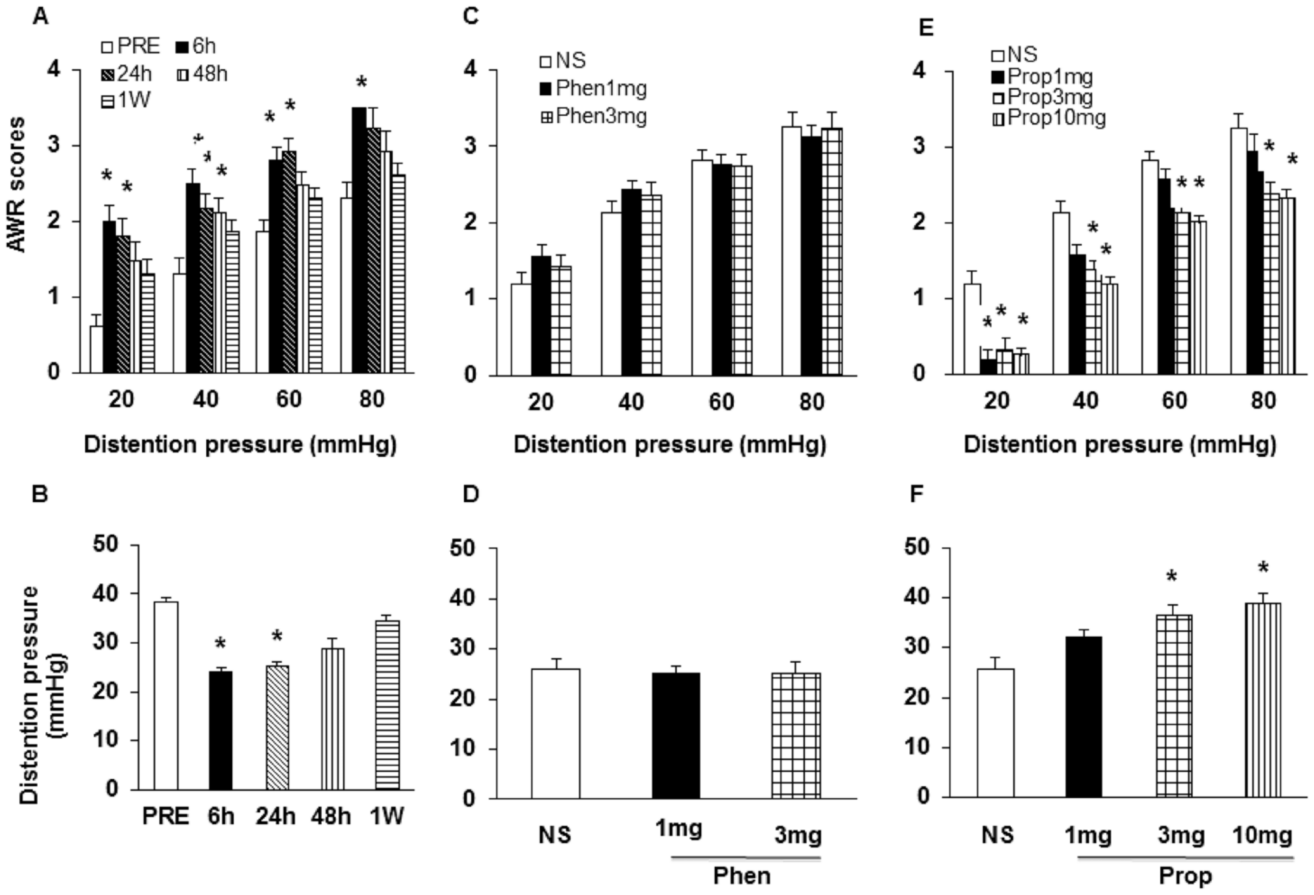
HIS-induced visceral hypersensitivity was reversed by β-adrenoceptor antagonist propranolol. (A) HIS significantly increased AWR scores in rats in response to CRD compared with pre-stress baseline (PRE). AWR scores started to increase at 6 hours and returned to normal level at 1week (1W) after HIS, *p<0.05 vs. PRE. (B) The distension threshold was reduced 6 and 24 hours after HIS and returned to pre-stress level at 48 hours after HIS, *p<0.05 vs. PRE. (C, D) The nonselective α-adrenoceptor antagonist phentolamine had no visible effect on the hypersensitivity induced by HIS. (E, F) The nonselective β-adrenoceptor antagonist propranolol reversed visceral hypersensitivity induced by HIS in a dose dependent manner, *p<0.05 vs. NS. NS: normal saline; Prop: propranolol; Phen: phentolamine.

In agreement with our previous report [6], [22], AWR scores were significantly increased at distention pressures of 20, 40, 60, 80 mmHg at 6 hours after HIS and kept high at pressures of 20, 40 and 60 mmHg at 24 hours after HIS (Fig. 1A, n = 8; *p<0.05 vs. PRE, Dunn's post hoc test following Friedman's ANOVA). AWR scores were significantly increased at distention pressures of 40 mmHg at 48 hours after HIS. Similarly, the distention threshold was dramatically decreased at 6 hours and 24 hours after end of the last stressor (Fig. 1B, n = 8; *p<0.05 vs. PRE, Dunn's post hoc test following Friedman's ANOVA). The distention threshold was returned to baseline levels at 48 hours and one week after end of the last stressor. To determine whether adrenergic signaling contributes to the induction of visceral hypersensitivity, rats subjected to HIS were treated once daily before each stress session with α-adrenoceptor antagonist phentolamine (Phen) or β-adrenoceptor antagonist propranolol (Prop). Normal saline treated rats served as controls (NS). Intraperitoneal (i.p.) administration of phentolamine had no visible effect on neither AWR scores nor distension threshold in HIS rats (Fig. 1C & D, n = 8). In contrast, administration of low dose of propranolol (1 mg/kg, i.p.) significantly decreased AWR scores at pressure of 20 mmHg at 6 hours after termination of the last stressor. Higher doses of propranolol (3 and 10 mg/kg) markedly decrease AWR scores at pressures of 20, 40, 60 and 80 mmHg at 6 hours after HIS (Fig. 1E, n = 8; *p<0.05 compared with NS, Tukey post hoc test following Kruskal-Wallis ANOVA). Similarly, the higher doses of propranolol (3 and 10 mg/kg) also dramatically increased distension threshold compared to NS (Fig. 1F, n = 8; *p<0.05, Tukey post hoc test following one way ANOVA). These data suggest that β-adrenoceptors are involved in HIS-induced visceral hypersensitivity.

### Butoxamine treatment alleviated HIS-induced visceral hypersensitivity

**Figure 2:**
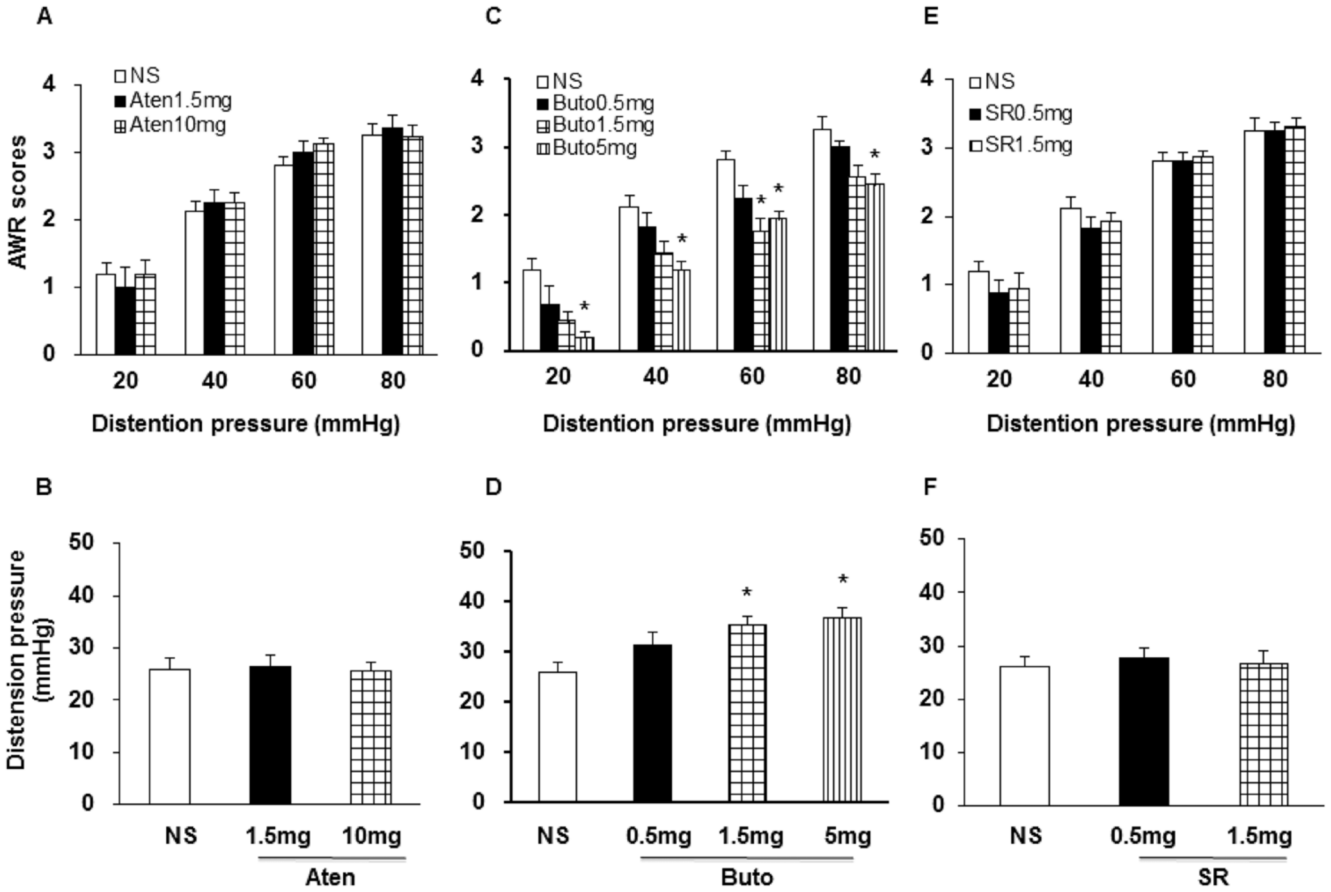
Administration of β_2_-adrenoceptor antagonist attenuated AWR scores in HIS rats. (A, B) The β_1_-adrenoceptor antagonist Atenolol (1.5, 10 mg/kg) had no visible effect on AWR scores and distension threshold. (C, D) The β_2_-adrenoceptor antagonist Butoxamine (0.5, 1.5 and 5 mg/kg) largely blocked visceral hyperalgesia induced by HIS in a dose-dependent manner, the maximal effect was observed at 5 mg/kg, *p<0.05 vs. NS. (E, F) The β_3_-adrenoceptor antagonist SR_59230A (0.5 and 1.5 mg/kg) had no visible effect on the visceral hypersensitivity induced by HIS. NS: normal saline; Buto: Aten: Atenolol; Butoxamine; SR: SR_59230A.

We next determined which subtypes of β-adrenoceptors are involved in HIS-induced visceral hypersensitivity. The β_1_-adrenoceptor antagonist atenolol (Aten, 1.5 and 10.0 mg/kg), β_2_-adrenoceptor antagonist butoxamine (Buto, 0.5, 1.5 and 5.0 mg/kg) or β_3_-adrenoceptor antagonist SR_59230A (SR, 0.5 and 1.5 mg/kg) was used in the present study (Fig. 2). Administration of β_2_-adrenoceptor antagonist butoxamine (i.p.) reduced AWR scores in HIS rats, in a dose-dependent manner (Fig. 2 C; n = 8; *p<0.05 vs. NS, Tukey post hoc test following Kruskal-Wallis ANOVA). Similarly, administration of Buto (1.5 and 5 mg/kg) also significantly increased distension threshold compared to NS (Fig. 2D, n = 8; *p<0.05, Tukey post hoc test following one way ANOVA). However, neither Atenolol (Fig. 2A & B; n = 8; Kruskal-Wallis ANOVA) nor SR_59230A (Fig. 2 E & F; n = 8; Kruskal-Wallis ANOVA) had significant effect on AWRs and distention threshold in HIS rats. These results provide evidence for β_2_-adrenergic receptor mediated signaling in HIS-induced visceral hypersensitivity.

### Butoxamine treatment reversed hyperexcitability of colon DRG neurons

**Figure 3:**
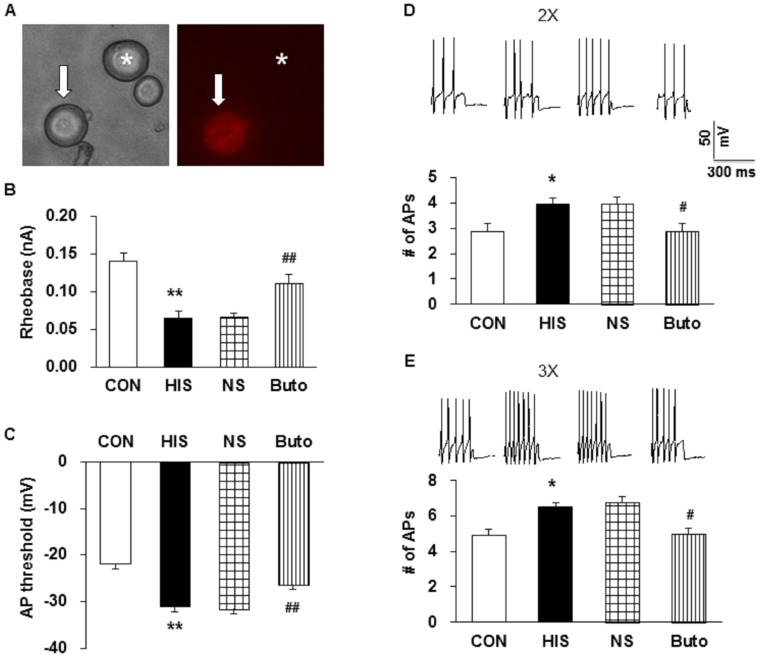
Administration of Butoxamine reversed hyperexcitability. (A) Patch clamp recording were made from DiI-labeled T_13_-L_2_ DRG neurons. An example of a DiI-labeled colon innervating DRG neuron is shown in the right panel (arrow). A phase image of the same DRG neuron is shown on the left panel (arrow). A star indicates a neuron without DiI labeling. (B) HIS treatment significantly reduced rheobase. **p<0.01 vs. control (CON). Butoxamine (Buto), which was intraperitoneally injected at the dose of 1.5 mg/kg body weight once a day for 9 days, significantly reversed the effect. ##p<0.01 vs. NS. (C) Graph showing depolarization of AP threshold after Buto treatment. **p<0.01 vs. CON; ##p<0.01 vs. NS. (D, E) Representative traces of APs induced by 300 ms depolarizing current pulses at 2 and 3 times rheobase were shown above. Bar graph showed that the average number of APs evoked by 2 and 3 times rheobase current was significantly reduced by Buto when compared with HIS group, *p<0.05 vs. CON; #p<0.05 vs. NS.

To determine the effect of butoxamine on excitability of colon innervating DRG neurons (including T13, L1 and L2 DRGs), we measured passive and active membrane properties of these neurons from control (CON), HIS, NS- or Buto-treated HIS rats. Figure 3A shows a DiI labeled neuron (Arrow) and a non-DiI labeled neuron (Star). Whole-cell patch clamping was employed to record DiI-labeled neurons. Rheobase, action potential (AP) threshold, and pattern of firing in response to depolarizing current stimulations were determined. Rheobase is the minimal injected current to evoke an AP. The respective average rheobase of colon innervating DRG neurons from HIS-treated animals were much lower than those from the corresponding controls (Fig. 3B, CON: 0.14±0.01 nA, HIS: 0.06±0.01 nA, n = 21 for each group, **p<0.01, vs. CON, Tukey post hoc test following Kruskal-Wallis ANOVA). Butoxamine treatment reversed the rheobase (Fig. 3B, NS: 0.07±0.00 nA; Buto: 0.11±0.01 nA, n = 21 for each group, ^##^p<0.01, vs. NS, Tukey post hoc test following Kruskal-Wallis ANOVA). AP threshold was significantly hyperpolarized by HIS and reversed by Butoxamine (Fig. 3C, CON: −21.9±2.52; HIS: −31.2±1.12; NS: −31.6±0.93; Buto: −26.5±1.22 mV, n = 21 for each group; ^###^p<0.01 vs. NS, Tukey post hoc test following Kruskal-Wallis ANOVA).

Number of APs in response to 2 times (2X) and 3 times (3X) rheobase stimulation was also recorded in colon innervating DRG neurons from HIS and butoxamine-treated group (Fig. 3D). The number of APs (2X) was 2.86±0.3 in control rats and 3.95±0.25 in HIS group. HIS significantly enhanced the number of APs evoked by 2X current stimulation (Fig. 3D, n = 21 for each group, *p<0.05 vs. CON, Tukey post hoc test following Kruskal-Wallis ANOVA). Similarly, HIS markedly enhanced the number of APs evoked by 3X current stimulation. The number of APs evoked by 3X current stimulation was 4.90±0.43 in control group and 6.48±0.39 in HIS group (Fig. 3E, n = 21 for each group, *p<0.05 vs. CON, Tukey post hoc test following Kruskal-Wallis ANOVA). Conversely, Buto treatment (i.p., 1.5 mg/kg) markedly reduced the number of APs evoked by 2X and 3X current stimulation. The number of APs evoked by 2X current stimulation was 2.90±0.3 in Buto group and 4.00±0.25 in NS group (Fig. 3D, n = 21 for each group, ^#^p<0.05 vs. NS, Tukey post hoc test following Kruskal-Wallis ANOVA). The number of APs evoked by 3X current stimulation was 5.00±0.4 in Buto group and 6.81±0.42 in NS group (Fig. 3E, n = 21 for each group, ^#^p<0.05 vs. NS, Tukey post hoc test following Kruskal-Wallis ANOVA).

**Figure 4:**
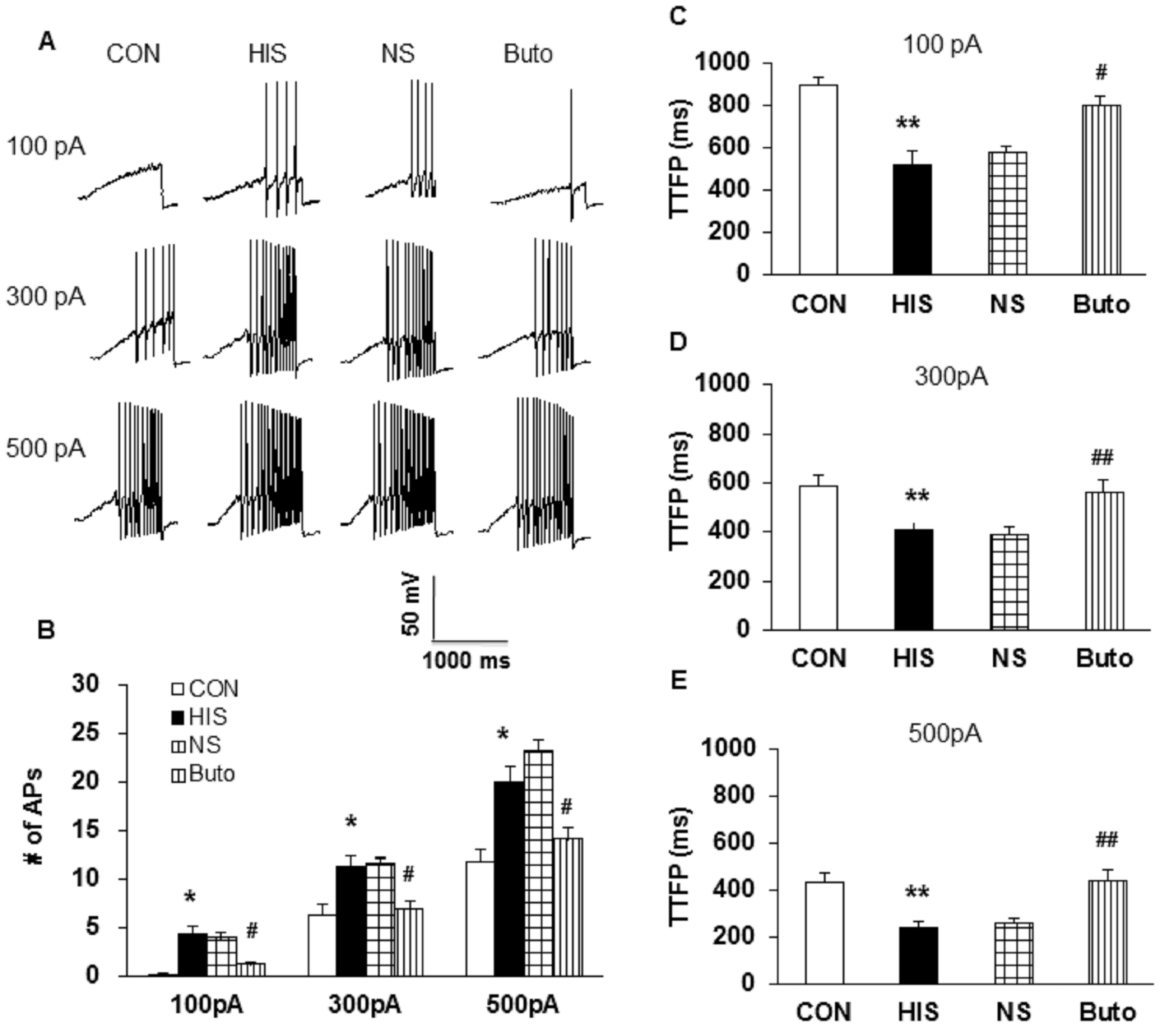
Administration of Butoxamine reduced number of APs induced by ramp current injection. (A) Representative traces of APs induced by a ramp current injection (100 pA, upper; 300 pA, middle; 500 pA, bottom) for control, HIS, NS- and Butoxamine-treated HIS rats were shown above. (B) Bar graph showed a significant decrease in number of APs in response to ramp stimulation after Butoxamine application, *p<0.05 vs. CON; #p<0.05 vs. NS. (C, D, E) Time to first peak (TTFP) was increased in HIS rats (**p <0.01 vs. CON) and reversed by Butoxamine (#p<0.05, ##p<0.01 vs. NS).

To further compare numbers of APs of colon DRG neurons after HIS and drug treatment, we also used 1-second ramp current stimulation ranging from 0 to 100 pA, 300 pA or 500 pA (Figure 4). HIS remarkably increased the number of APs in response to a 100 pA, 300 pA and 500 pA ramp current stimulation when compared with controls (Fig. 4A and B). The number of firings in responding to 100 pA ramp stimulation was 0.24±0.12 in control group and was 4.29±0.82 in HIS group (Fig. 4A and B, n = 21 for each group, *p<0.05 vs. CON, Tukey post hoc test following Kruskal-Wallis ANOVA). The number of firings in response to 300 pA ramp stimulation was 6.33±1.06 in control group and was 11.29±1.15 in HIS group (Fig. 4A and B, n = 21 for each group, *p<0.05 vs. CON, Tukey post hoc test following Kruskal-Wallis ANOVA). The number of firings in responding to 500 pA ramp current stimulation was 11.71±1.42 in control rats and 20.00±1.54 in HIS group (Fig. 4A and B, n = 21 for each group, *p<0.05 vs. CON, Tukey post hoc test following Kruskal-Wallis ANOVA). In addition, the time to first spike (TTFS) in response to a 100 pA, 300 pA and 500 pA current injection was analyzed in the present study. HIS significantly decreased TTFS (Fig. 4C, 100 pA, CON: 895.3±41.06 ms, HIS: 521.6±63.78 ms, **p<0.01; Fig. 4D, 300 pA, CON: 583.9±46.69 ms, HIS: 406.4±37.36 ms, **p<0.01; Fig. 4E, 500 pA, CON: 436.4±36.55 ms, HIS: 244.02±21.62 ms, **p<0.01). These data suggest an effect of HIS on spike generation of colon specific DRG neurons. Conversely, Buto treatment markedly reduced the number of APs evoked by various ramp current stimulations (Fig. 4A and B). The number of firings in responding to 100 pA ramp stimulation was 3.95±0.64 in NS group and 1.19±0.36 in Buto group (Fig. 4B, n = 21 for each group, ^#^p<0.05 vs. NS, Tukey post hoc test following Kruskal-Wallis ANOVA). The number of repetitive firing in response to 300 pA ramp stimulation was 11.57±0.76 in NS group and was 6.86±1.02 APs in Buto group (Fig. 4B, n = 21 for each group, ^#^p<0.05 vs. NS, Tukey post hoc test following Kruskal-Wallis ANOVA). The number of firings in responding to 500 pA ramp current stimulation was 23.14±1.21 in NS group and 14.14±1.21 in Buto group (Fig. 4B, n = 21 for each group, ^#^p<0.05 vs. NS, Tukey post hoc test following Kruskal-Wallis ANOVA). In addition, the TTFS in response to 100 pA, 300 pA and 500 pA current injections was significantly increased after Buto treatment (Fig. 4C, 100 pA, Buto: 802.0±39.59 ms, NS: 580.6±28.08 ms, ^#^p<0.05; Fig. 4D, 300 pA, Buto: 567.3±47.52 ms, NS: 393.4±25.49 ms, ^##^p<0.01; Fig. 4E, 500 pA, Buto: 445.6±43.63 ms, NS: 265.6±16.89 ms, ^##^p<0.01). These data suggest an effect of Buto on spike generation of colon specific DRG neurons of HIS rats.

### Propranolol treatment increased sustained potassium current density

**Figure 5:**
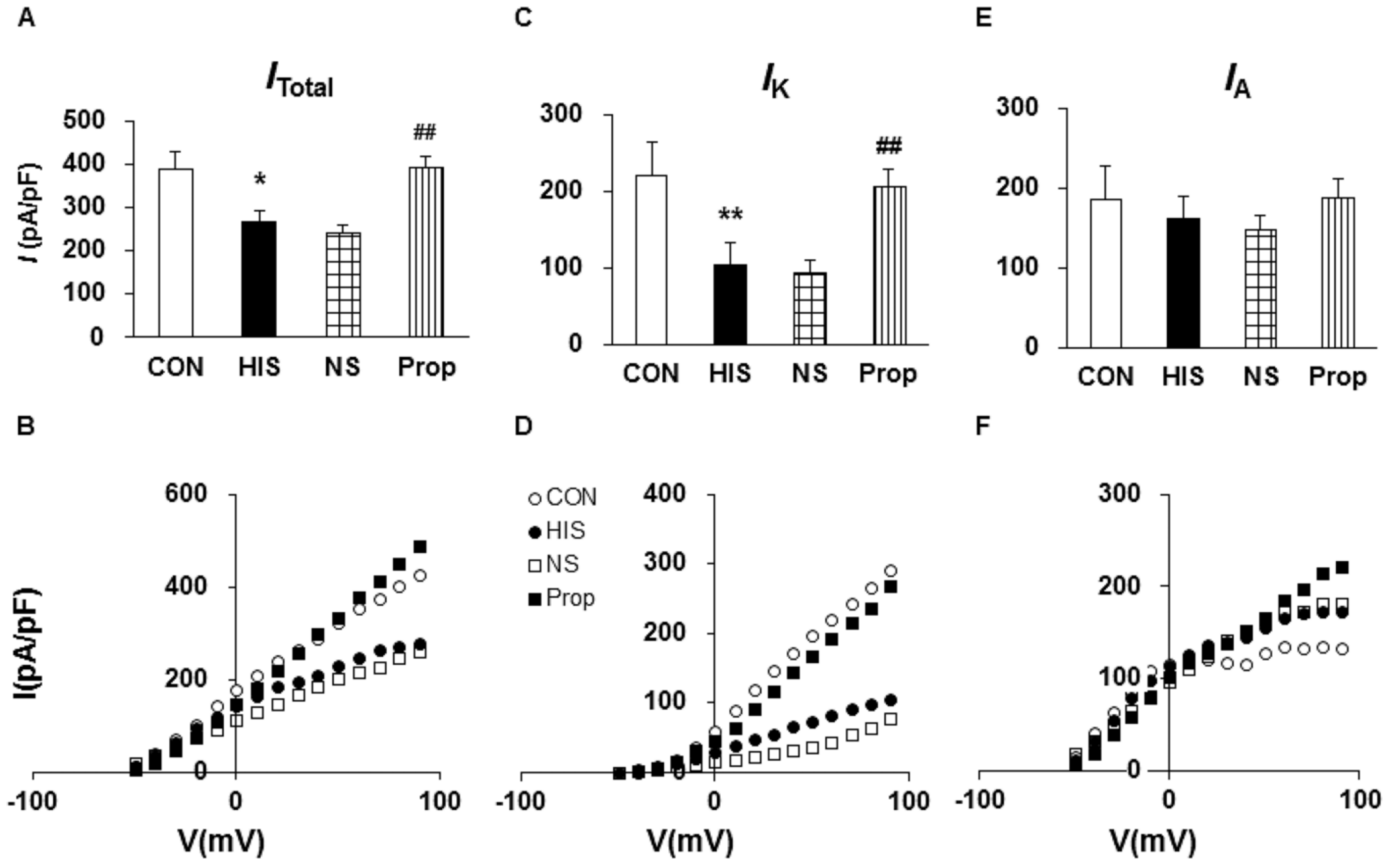
Administration of propranolol increased voltage-gated potassium current. Currents were measured at different holding potentials. For recording total voltage-gated potassium current (*I*_Total_), the membrane potential was held at −100 mV and voltage steps were from −50 to +90 mV with10-mV increments and 400 ms duration. For recording the sustained K_V_ current (*I*_K_), the membrane potential was held at −50 mV and the voltage steps were the same as above. Currents generated by these two protocols were subtracted to produce *I*_A_. Bar graphs showed the mean peak *I*_Total_ (A), *I*_K_ (C), and *I*_A_ (E) densities from control (CON), HIS, NS- and Prop-treated HIS (Prop) rats. The current density (pA/pF) was calculated by dividing the current amplitude by cell membrane capacitance. (A) HIS produced a significant reduction in *I*_Total_ while Prop treatment caused a significant increase in *I*_Total_ (*p<0.05, compared with CON, two sample t-Test; ##p<0.01, compared with NS). (B) HIS led to a significant decrease in *I*_K_ while administration of Prop resulted in a marked potentiation in *I*_K_ density (*p<0.05, compared with CON, two sample *t*-Test; ##p<0.01, compared with NS). (E) The *I*_A_ density was not significantly altered after HIS and Prop treatment. (B, D, F) Current (*I*)-Voltage (V) curves for *I*_Total_ (B), *I*_K_ (D), and *I*_A_ (F) recorded from control, HIS, NS- and Prop-treated HIS rats. HIS treatment did not significantly change the reversal potentials of these currents.

Since changes in activation thresholds and spike frequency suggest that there is an alteration in voltage-gated potassium (K_V_) channels, we next performed patch-clamp recordings to examine K_V_ currents under voltage-clamp conditions. As described previously [20], Na^+^ in the normal external solution was replaced with equimolar choline. Ca^2+^ concentration was reduced to 30 µM. A depolarization step from −50 to +90 mV in 10-mV increments with duration of 400 ms activated all K_V_ channels (*I*_Total_; Fig. 5A and B). The peak current-voltage (*I*–V) curves are shown in Figure 5B. However, HIS treatment greatly decreased peak current density in colon DRG neurons (Fig. 5A, *p<0.05, compared with CON, Tukey post hoc test following one-way ANOVA). The mean peak current density of *I*_Total_ was 388.45±42.50 pA/pF (n = 8) from CON rats and 265.41±28.06 pA/pF (n = 8) from HIS rats. Because there were two main types of K_V_ currents (*I*_A_ and *I*_K_) described in nociceptive DRG neurons [20], [26], [27], we then isolated these two K_V_ currents by manipulating the holding membrane potential. A depolarization step −50 to +90 mV with 400 ms in duration in 10-mV increments activated most of the sustained K_V_ channels (Fig. 5C and D) but not A-type K_V_ channels. Subtraction of *I*_K_ from *I*_Total_ yielded *I*_A_ (Fig. 5E and F). In the present study, the mean peak current density of *I*_K_ was 221.15±27.29 pA/pF (n = 8) from control rats and 104.76±8.26 pA/pF from HIS rats (n = 8). *I*_K_ density was markedly reduced following HIS (Fig. 5C, **p<0.01, compared with CON, Tukey post hoc test following one-way ANOVA). However, the *I*_A_ density was not significantly changed (Fig. 5E, p>0.05, compared with CON, Tukey post hoc test following one-way ANOVA). The mean peak current density of *I*_A_ was 167.30±20.38 pA/pF (n = 8) from control rats and 160.65±25.66 pA/pF from HIS rats (n = 8). These data suggest an involvement of *I*_K_ in the development of visceral hypersensitivity induced by HIS in rats.

In an effort to investigate the contribution of β-adrenoceptor mediated signaling, propranolol was used in this experiment. Administration of propranolol (i.p.) significantly enhanced the *I*_Total_ (Fig. 5 A and B) and *I*_K_ density (Fig. 5 C and D) without any significant alteration in *I*_A_ density (Fig. 5 E and F) when compared with NS-treated rats. The mean peak current density of *I*_Total_ was 241.72±16.94 pA/pF (n = 8) from NS rats and 394.12±23.57 pA/pF (n = 8) from Prop-treated rats. The mean peak current density of *I*_K_ was 93.79±9.19 pA/pF (n = 8) from NS rats and 206.18±19.42 pA/pF from Prop-treated rats (n = 8). The mean peak current density of *I*_A_ was 147.93±13.44 pA/pF (n = 8) from NS rats and 187.94±21.60 pA/pF from Prop-treated rats (n = 8). These data suggest an involvement of β-adrenoceptor mediated signaling in the suppression of *I*_K_ induced by HIS in rats.

### Administration of NE enhanced neuronal excitability of colon-specific DRG neurons

**Figure 6:**
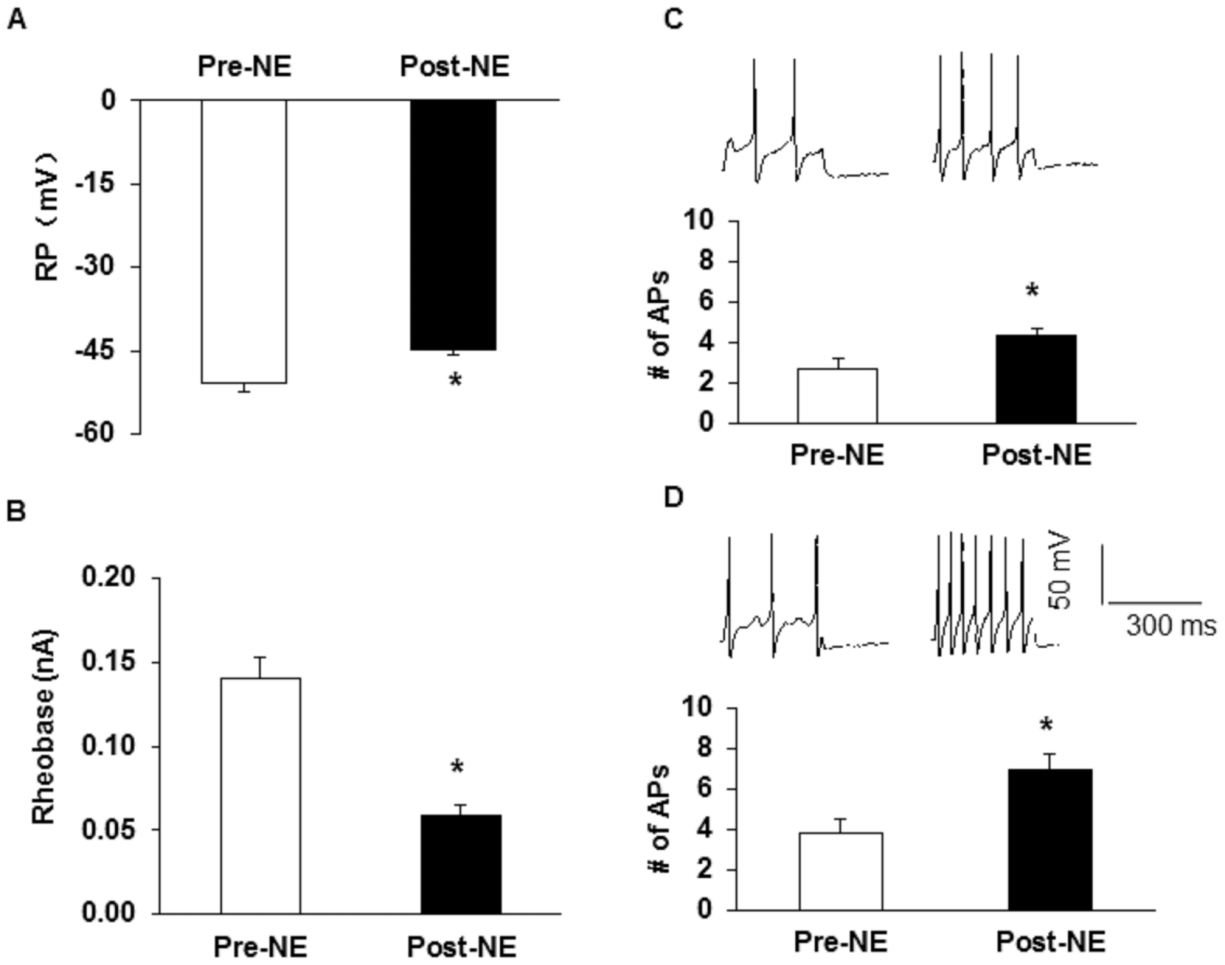
Incubation of NE enhanced excitability of colon-specific DRG neurons. (A) NE perfused for 3 min significantly depolarized the resting membrane potentials. *p<0.05 vs. Pre-NE group. (B) NE application markedly reduced rheobase. *p<0.05 vs. Pre-NE group. (C, D) Representative traces of action potentials (APs) induced by 2 and 3 times rheobase current injection. Bar graph showed a significant increase in number of APs after NE application, *p<0.05 vs. pre-NE group.

**Figure 7:**
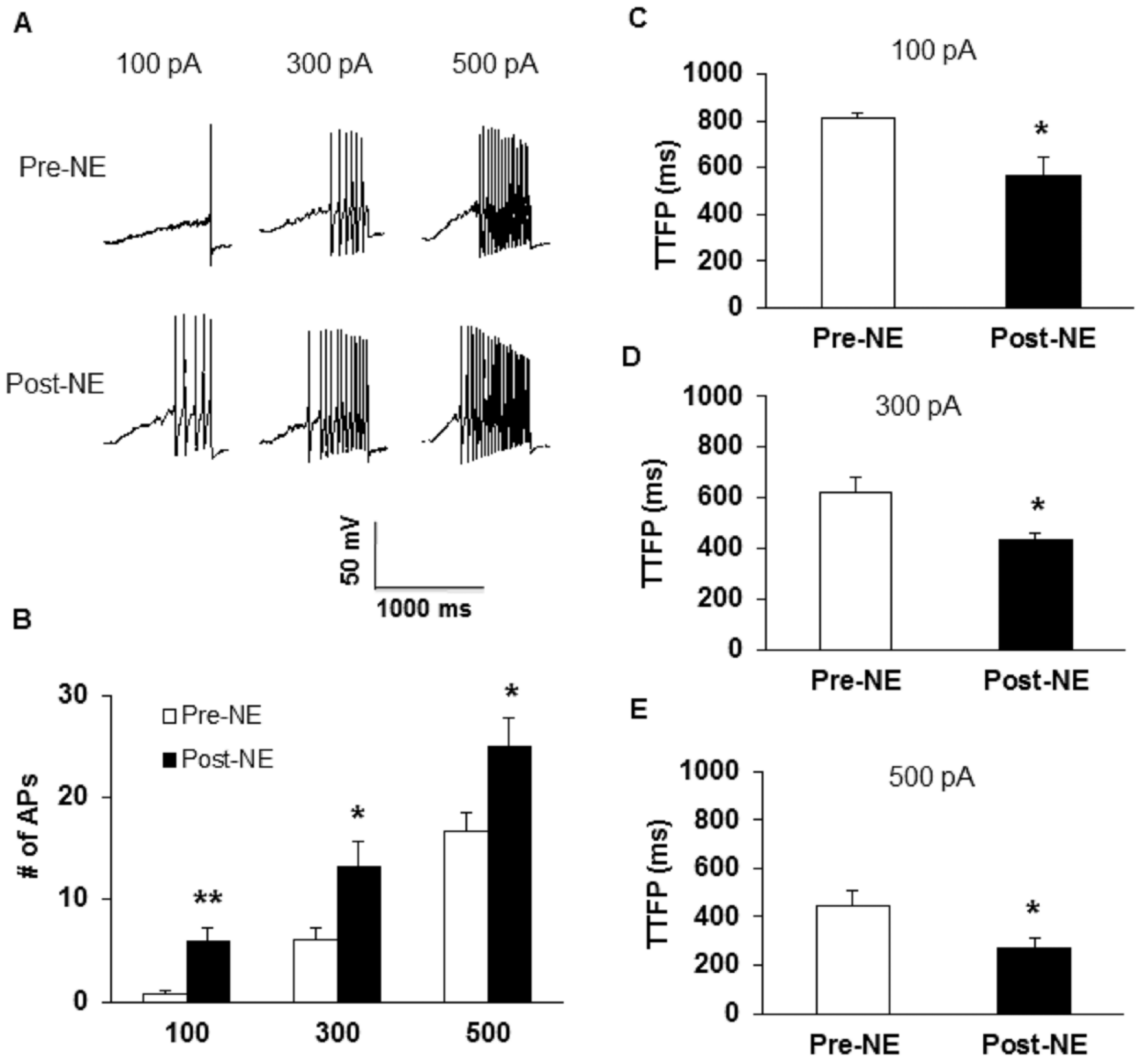
Incubation of NE increased number of AP evoked by ramp current stimulation. (A) Representative traces of APs induced by a ramp current injection (left, 100 pA; middle, 300 pA; right, 500 pA) before (Pre) and after (Post) NE application. (B) Bar graph showed a significant increase in the number of APs in response to ramp stimulations after NE application, *p<0.05 **p<0.01 vs. pre-NE. (C, D, E) Time to first peak (TTFP) of AP in response to 100 pA (C, *p<0.05, compared with Pre), 300 pA (D,*p <0.05, compared with Pre) and 500 pA ramp current stimulation (E, *p<0.05, compared with Pre) was significantly decreased after NE incubation.

To further confirm the role of adrenergic signaling, we examined whether NE can increase neuronal excitability of colon innervating DRG neurons of healthy control rats. NE was freshly prepared at the concentration of 10 µM and added into the recording chamber for 3 minutes. Incubation of colon DRG neurons with NE significantly depolarized the resting membrane potential (Fig. 6A, Pre: −51±1.56 mV; Post: −45±0.90 mV; n = 15; *p<0.05, paired sample sign test) and reduced rheobase (Fig. 6B, Pre: 0.14±0.01 nA; Post: 0.059±0.01 nA; n = 15; *p<0.05, paired sample t-Test). Application of NE also increased the number of APs evoked by 2X and 3X rheobase current stimulation (2X, Fig. 6C, Pre: 2.67±0.49, Post: 4.27±0.42, n = 15, *p<0.05, paired sample sign test; 3X, Fig. 6D, Pre: 3.80±0.71; Post: 6.93±0.77; n = 15, *p<0.05, paired sample sign test). In addition, the number of APs in response to a 100, 300 and 500 pA ramp current stimulation was also remarkably increased in after NE incubation when compared with Pre-NE group (Fig. 7A and B, for 100 pA, Pre: 0.75±0.31, Post: 5.83±1.42, n = 6, **p<0.01, paired sample t-test; for 300 pA, Pre: 6.00±1.17, Post: 13.17±2.56, n = 6, *p<0.05, paired sample sign test; for 500 pA, Pre: 16.75±1.82, Post: 25.00±2.85, n = 6, *p<0.05, paired sample t-test). NE treatment also significantly decreased the TTFP in response to a 100 pA (Fig. 7C, paired sample t-test), 300 pA (Fig. 7D, paired sample t-test) and 500 pA (Fig. 7E, paired sample t-test) ramp current injection. These data suggest that NE treatment enhanced the excitability of colon innervating DRG neurons of healthy control rats.

### Intrathecal injection of NE produced visceral hypersensitivity

**Figure 8:**
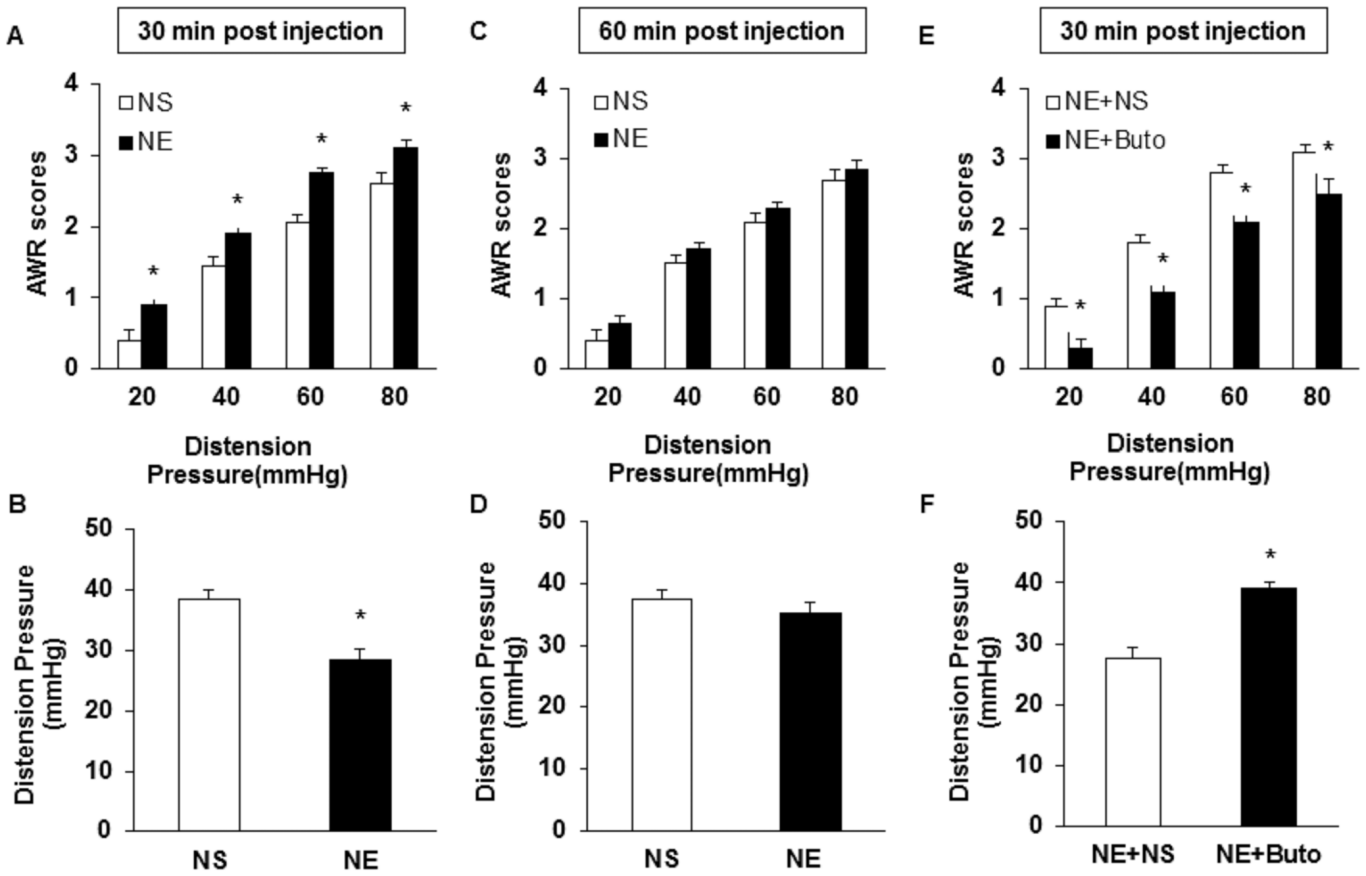
Visceral hypersensitivity induced by NE. (A) Application of NE (i.t.) significantly increased AWR scores in healthy control rats 30 min after intrathecal injection (n = 10, *p<0.05 vs. NS). (B) Application of NE significantly reduced distention threshold when tested 30 min after intrathecal injection (n = 10, *p<0.05 vs. NS). (C, D) The AWRs and distention threshold returned to normal level 60 min after the injection (n = 10 for each group). (E, F) Butoxamine administration (i.p.) blocked the effect of NE (i.t.) in healthy control rats (n = 5 for each group, *p<0.05 vs. NE+NS).

If NE generated endogenously contributes to the initiation and maintenance of visceral hypersensitivity in HIS rats, then application of exogenous NE to healthy rats should mimic the effects of HIS. In the present study, NE was intrathecally applied to healthy rats and behavioral responses were assessed. NE administration produced a significant increase in AWR scores at pressures of 20, 40, 60 and 80 mmHg 30 minutes after the injection (Fig. 8A, n = 10 for each group; *p<0.05 vs. NS group, Mann-Whitney test) and a significant decrease in distention threshold (Fig. 8B, n = 10 for each group, *p<0.05 vs. NS group, Mann-Whitney test). The AWRs and distention threshold returned to normal level 60 min after the injection (Fig. 8C and D, n = 10 for each group). Butoxamine administration blocked the NE effect (Fig. 8E and F, n = 5 for each group; *p<0.05 vs. NE group, Mann-Whitney test). These data indicate that NE produced acute hyperalgesic effect in healthy rats, which was blocked by a β_2_-adrenoceptor antagonist.

### HIS resulted in an elevation of NE concentration without alteration in expression of β_2_-adrenoceptors

**Figure 9:**
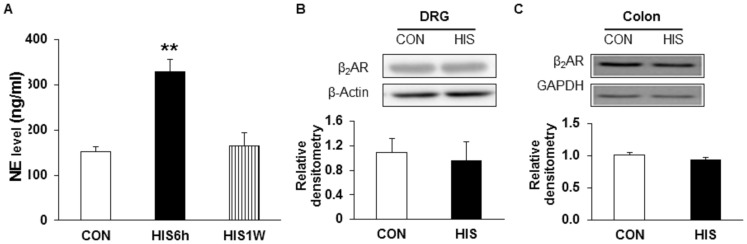
Elevation of NE concentration in blood plasma in HIS rats. (A) The concentration of NE in blood plasma was dramatically increased when tested at 6 hours after the last stressor and returned to control level when tested 1 week after termination of the last stressor (**p<0.01 vs. con n = 4 for control and 6 h group, n = 3 for 1 week group). (B) Expression of β_2_-adrenoceptors in T_13_-L_2_ DRGs was not changed 6 hours after HIS (n = 4 for each group). (C) Expression of β_2_-adrenoreceptors in the muscularis exerna of the colon was not changed 6 hours after HIS (n = 4 for each group).

We next measured the NE concentration in the blood plasma after HIS. The plasma concentration of NE significantly increased 6 hours after termination of the last stressor and return to base level 1 week after the last stressor (Fig. 9A, n = 4 for control and 6 hour group, n = 3 for 1 week group; **p<0.01 vs. CON, Tukey post hoc test following Kruskal-Wallis ANOVA). To determine whether expression of β_2_-adrenoceptor increases in T_13_-L_2_ DRGs and in the muscularis externa of the distal colon after HIS, western blotting assay was performed. Anti-β_2_-adrenoceptor antibody labeled a 55 kDa molecular weight protein. Expression of β_2_-adrenoceptors in the DRGs 6 hours after end of the last stressor was not significantly changed when compared with controls (Fig. 9B, n = 4 for each group, two sample t-test). In addition, expression of β_2_-adrenoceptors in the muscularis externa of the distal colon 6 hours after end of the last stressor was not significantly changed when compared with controls (Fig. 9C, n = 4 for each group, two sample t-test).

## Discussion

In the present study, HIS induced visceral hypersensitivity to CRD lasted for 24 hours and returned to baseline by 48 hours in male SD rats after termination of the last stressor, which is consistent with our previous study [22]. By contrast, Winston et al. reported that HIS induced visceral hypersensitivity to CRD that lasted for at least 8 hours, and returned to baseline by 24 hours in male Wistar rats [6], indicating the mechanisms by which chronic stress leading to colonic dysfunction differs by animal species. Winston et al. reported that blockade of adrenergic signaling with α_1_/α_2_- and β_1_/β_2_-adrenoceptors before the daily application of stress prevented the induction of visceral hypersensitivity, but they didn't identify which type of adrenoceptors mainly mediates the prolonged induction of hypersensitivity in their model [6]. Our data provided additional evidence to confirm the idea that β_2_-adrenoceptor plays an important role in chronic stress-induced visceral hypersensitivity. This conclusion is based on the following observations. Firstly, we showed that HIS-induced visceral hypersensitivity was attenuated by non-selective β-adrenoceptor antagonist propranolol but not by non-selective α-adrenoceptor antagonist phentolamine (Fig. 1). Secondly, butoxamine, a selective β_2_-adrenoreceptor antagonist, significantly reversed visceral hyperalgesia in HIS-treated rats, in a dose-dependent manner (Fig. 2) while the selective β_1_- and β_3_-adrenoceptor antagonist did not produce any effect on visceral hyperalgesia in HIS-treated rats. Finally, nonselective antagonist of β_2_-adrenoceptor didn't produce any obvious effect in healthy control rats (data not shown), suggesting that butoxamine-induced effect was not a non-specific analgesic effect. This also suggested that the role of β_2_-adrenoceptor in signaling colonic distension may not be as important in healthy as in the sensitized state.

In addition to antagonism on pain behaviors, butoxamine treatment remarkably reduced excitability of colon innervating DRG neurons (Figs. 3 and 4). In agreement with our previous study [6], HIS significantly enhanced the excitability of colon specific DRG neurons, as was evidenced by our findings that HIS markedly reduced the rheobase and increased number of APs evoked by 2 and 3 times current stimulation, and by various amounts of ramp current stimulation. After butoxamine treatment, the rheobase was greatly increased and the number of APs evoked by 2 and 3 times current stimulation, and by 100, 300 and 500 pA ramp current stimulation was significantly decreased. Additionally, the time to first peak was also reversed in butoxamine-treated HIS rats. This raised a possibility that functions of voltage-gated Na^+^ and/or K^+^ channels may be modified following butoxamine treatment because these channels are major determinant factors for rheobase and repetitive firings. The present study showed that application of β-adrenoceptor antagonist significantly reversed the suppression of *I*_K_ density caused by HIS in T_13_-L_2_ DRGs (Fig. 5). The reduction in *I*_K_ density induced by HIS may be a underlying mechanism for the enhanced excitability of DRG neurons, while the increase in *I*_K_ density by application of β-adrenoceptor antagonist may well contribute to normalized excitability of DRG neurons. In addition to the effect of *I*_K_, the contribution of *I*_A_ was also considered since *I*_A_ has been reported to be one of the major players in control of neuronal excitability [26], [28]. However, *I*_A_ was not altered by HIS and propranolol in the present study. It is more likely that HIS plays a specific effect on *I*_K_. In addition to the suppression of *I*_K_, the present study cannot exclude the role of Na_V_ channels since an increase in *I*Na_V_ also contributed to the increased excitability of T_13_-L_2_ DRGs by HIS [22]. We have previously reported that the expression of cystathionine beta-synthetase (CBS) was upregulated by HIS [22] and that hydrogen sulfide (H_2_S), the product of CBS, suppresses *I*_K_ of trigeminal neurons [29]. Further studies are warranted to determine the relationship between H_2_S pathway and adrenergic signaling and their roles in the modulation of *I*_K_ in HIS model.

Another important finding is that HIS elevated the NE concentration in blood plasma without alteration of the expression of β_2_-adrenoreceptor. NE is one the most prominent mediators of stress response [30]. We measured the plasma concentration of NE to determine whether NE is a candidate to induce visceral hypersensitivity. In a line with previous report [5], the blood plasma level of NE remarkably increased by about 2-fold 6 hours after end of the last stressor. To further confirm the role of NE in the development of visceral hypersensitivity, additional experiments were performed. Firstly, i.t. injection of NE produced visceral hypersensitivity of healthy rats, which mimics the effect of HIS on visceral sensitivity. Secondly, incubation of colon innervating DRG neurons *in vitro* with NE greatly enhanced the excitability (Fig. 6). This conclusion was supported by our observations that NE significantly decreased the rheobase, increased the number of APs evoked by 2 and 3 times current stimulation, and by various ramp current stimulation. Together, these data suggest that NE mimics the effect of HIS on neuronal excitability. Of note is that the mechanism by which NE and HIS enhanced neuronal excitability and visceral hypersensitivity might differ to some degree. The acute application of NE is unlikely to increase channel expression. HIS, however, significantly enhanced expression of Nav 1.7 and Nav 1.8 of T_13_-L_2_ DRGs [22]. NE elevated in the blood plasma and the colon wall could increase the expression of nerve growth factor in the colon wall, thus sensitizing primary afferents [6]. Nevertheless, our findings indicate a crucial role for NE signaling in the development of visceral hypersensitivity and in the hyperexcitability of colon-specific DRG neurons. The mechanism underlying the elevation of NE level is not clear. Several studies suggest that the hypothalamic-pituitary-adrenal axis and the sympathetic system originating in the locus coeruleus are responsible for the elevated NE level [6], [31]. It is also possible that NE reuptake transporter (NET) might play a role since NET was reported to be downregulated by chronic-acute combined stress [8]. Although the detailed mechanisms for an increase in NE concentration have yet to be investigated, our findings indicate that enhanced NE concentration might be the major contributor to visceral hypersensitivity following HIS.

Since only β_2_-adrenergic receptor antagonist attenuates the visceral hypersensitivity and reverses the neuronal hyperexcitability, we therefore assayed expression of β_2_-adrenergic receptors in rat DRGs and the colon after HIS. β_2_-adrenoceptors have been reported to exist widely both in central and in peripheral nervous system [32]–[38]. Since butoxamine could not cross blood brain barrier [39], the present study suggested an important role of peripheral β_2_-adrenoceptor activation in the development of HIS-induced visceral hypersensitivity. In an effort to detect expression of β_2_-adrenoceptors, we showed that expression of β_2_-adrenoceptors was not altered in colon DRGs. It is possible that the expression of β_2_-adrenoceptors was not altered in DRGs, where the cell bodies locate, because β_2_-adrenoceptors after expression are transported to the peripheral terminals of DRGs. Therefore, we detected the expression of β_2_-adrenoceptors in the muscularis externa of the distal colon. We further confirm that the expression of β_2_-adrenoceptors in the muscularis externa of the colon was not altered. It is expected that function of β_2_-adrenoceptors would be enhanced in colon DRGs. Further experiments are warranted to investigate whether the function of β_2_-adrenergic receptors is enhanced after HIS.

In summary, these data provide evidence that NE elevated in blood plasma activates β_2_-adrenoreceptor to enhance the excitability of colon-specific DRG neurons, thus producing visceral hypersensitivity; Blockade of β_2_-adrenoreceptor reverses hyperexcitability and attenuates visceral hypersensitivity to colorectal distension after HIS. These findings suggest that β_2_-adrenoreceptor may be a potential target for novel agents for the treatment of visceral pain in patients with IBS and related disorders.
